# Ground-glass nodule in a patient with echinoderm microtubule-associated protein-like 4-anaplastic lymphoma kinase (EML4-ALK)-positive lung cancer: a case report

**DOI:** 10.1186/s12957-016-0841-5

**Published:** 2016-03-10

**Authors:** Yuki Owada, Atsushi Yonechi, Mitsunori Higuchi, Hiroyuki Suzuki

**Affiliations:** Department of Thoracic Surgery, Takeda General Hospital, Fukushima, Japan; Department of Regenerative Surgery, School of Medicine, Fukushima Medical University, 1 Hikarigaoka, Fukushima, 960-1295 Japan

**Keywords:** Lung cancer, EML4-ALK-positive, Ground-glass nodule, Surgery

## Abstract

**Background:**

Grand-glass nodule for CT image has thought to be less aggressive tumor in lung cancer. Echinoderm microtubule-associated protein-like 4-anaplastic lymphoma kinase (EML4-ALK)-positive lung cancer presenting with Ground-glass nodules (GGNs) is relatively rare, and few such cases have been reported.

**Case presentation:**

An asymptomatic 56-year-old woman exhibited a 1.1-cm GGN in the lower lobe of the left lung on computed tomography during a medical checkup. Positron emission tomography showed no difference in uptake by the nodule compared with other organs. We elected to perform surgery because the nodule included a solid component and had grown only slightly during the last 2 years according to thin-section computed tomography. Partial resection of the lower left lung was performed by video-assisted thoracic surgery. Pathological examination revealed mucus-producing high columnar epithelium forming an irregular tubular-acinar-like structure partly replacing the alveolar epithelium on hematoxylin and eosin staining. More than 50 % of the tumor demonstrated a lepidic growth pattern. The tumor was negative for epidermal growth factor receptor mutation but positive for the EML4-ALK fusion oncogene according to fluorescence in situ hybridization.

**Conclusions:**

We herein report a case of EML4-ALK-positive lung cancer presenting with a GGN along with a review of the relevant literature, including histopathological findings and imaging features. We consider that EML4-ALK-positive lung cancer is often highly progressive and that careful follow-up is therefore essential in these patients.

## Background

Almost all grand-glass nodules for computed tomography (CT) image have thought to be less aggressive tumor in lung cancer. However, we find that there are a few GGN that would potentially be a progressive type. Here, we show a case of lung cancer with EML4-ALK positive that shows GGN for CT with potentially aggressive.

## Case presentation

**Fig. 1:**
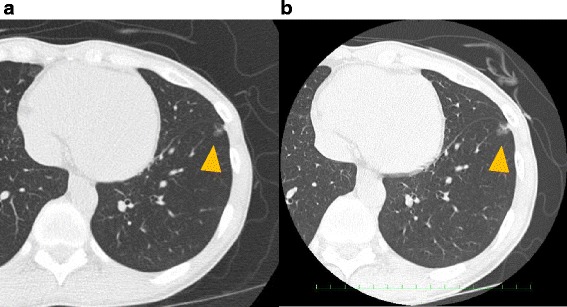
Thin-section CT showed a GGN with a small solid component in the left lower lung. CT during a medical checkup showed a nodule. Follow-up CT 2 years after the medical checkup showed that the nodule had grown slightly. Yellow allowheads indicate the location of tumor

**Fig. 2:**
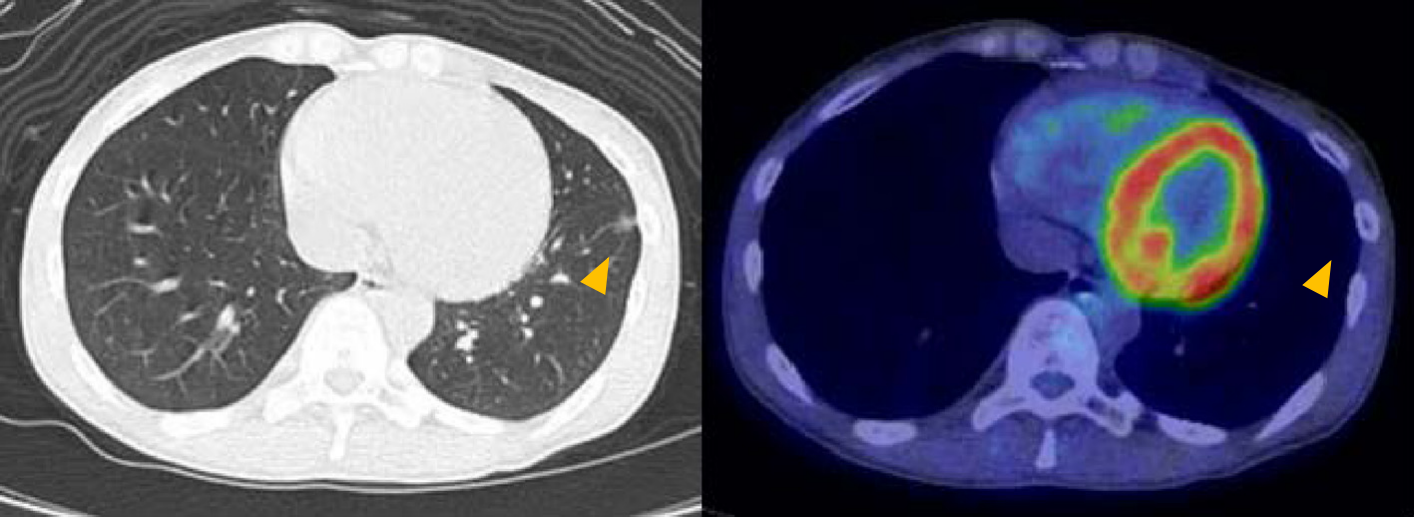
Positron emission tomography-CT showed no abnormal accumulation in the nodule in the left lower lung. Yellow allowheads indicate the location of tumor

**Fig. 3:**
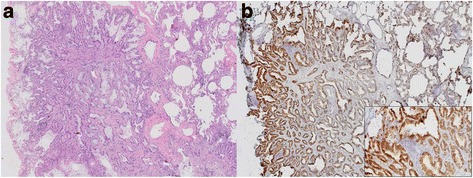
Histopathological observations. **a** Hematoxylin and eosin staining of the tumor. Mucus-producing high columnar epithelium formed an irregular tubular-like or acinar-like structure that replaced the alveolar epithelium. **b** ALK immunostaining of the tumor showed an immunohistochemical iScore of 3 (0 = no stained cells; 1 = 0–50 % stained tumor cells; 2 = 50–80 % stained tumor cells or >80 % stained tumor cells with marked variability of staining intensity; 3 = >80 % stained tumor cells without marked variability of staining intensity)

An asymptomatic 56-year-old woman with no smoking history exhibited a 1.1-cm ground-glass nodule (GGN) in the lower lobe of the left lung on CT during a medical checkup (Fig. 1a). Positron emission tomography showed no difference in uptake by the nodule compared with other organs (Fig. 2). All tumor markers were within the normal range. Over 2 years, the size of the nodule had grown from 0.9 to 1.1 cm according to thin-section CT (Fig. 1b); thus, the patient was clinically suspected of having lung cancer. Transbronchial lung biopsy was not performed before surgery because the nodule was small and peripherally located. We elected to perform surgery for both diagnosis and treatment. Partial resection of the lower left lung was performed by video-assisted thoracic surgery. The tumor with a slight change in the visceral pleura was confirmed macroscopically. We got a judgement of a not invasive tumor by intraoperative pathological diagnosis; thus, lymph node excision has not been done. Finally, pathologically, the tumor was diagnosed as an adenocarcinoma, and mucus-producing high columnar epithelium formed an irregular tubular-acinar-like structure partly replacing the alveolar epithelium on hematoxylin and eosin staining (Fig. 3a). More than 50 % of the tumor demonstrated a lepidic growth pattern, while the remainder showed an invasive adenocarcinoma structure. Accordingly, the tumor was diagnosed as type C according to the Noguchi classification. The tumor was negative for epidermal growth factor receptor mutation but positive for the echinoderm microtubule-associated protein-like 4-anaplastic lymphoma kinase (EML4-ALK) fusion oncogene according to fluorescence in situ hybridization (Fig. 3b). Finally, the pathological stage of lung cancer was determined to be T1aN0M0 stage 1A. The patient was discharged on postoperative day 5 and showed no signs of postoperative complications or recurrence 13 months after surgery.

### Discussion

The EML4-ALK fusion oncogene is present in 3.7 to 6.8 % of patients with lung adenocarcinomas [1, 2]. Few reports have discussed the imaging features of EML4-ALK-positive lung cancer, although many such tumors present as solid masses. The CT findings in patients with EML4-ALK-positive lung cancer are shown in Table 1.

**Table 1:** Previous CT imaging findings in patients with ALK-positive lung cancer

Author (year)	Park (2014)	Fukui (2012)	Kikuchi (2014)
Number of ALK+ lung cancer	36 %	28 %	28 %
With GGN	1 (2.8)	1 (3.6)	1 (20.0)
Spiculation	N/A	N/A	1 (20.0)
Notch	N/A	N/A	N/A
Air bronchogram	N/A	N/A	4 (80.0)
Pleural indentation	N/A	N/A	2 (40.0)
Pleural effusion	15 (41.7)	N/A	N/A
Intrapulmonary metastasis	N/A	N/A	N/A
Lymphadenopathy	31 (86.1)	N/A	N/A
Extended lymph node invasion	7 (19.4)	N/A	N/A
Lymphangitis	3 (8.3)	N/A	N/A

*N*/*A* not applicable

EML4-ALK-positive lung cancer presenting with GGN is relatively rare. To the best of our knowledge, only one previous case report of a patient with EML4-ALK-positive lung cancer presenting with GGN has been published [3]. In another study that examined lesions in 104 patients with a ≥50 % GGN component on thin-section CT, EML4-ALK positivity was only observed in 3 % of cases, whereas epidermal growth factor receptor (EGFR) mutation was observed in 64 % [4]. Park et al. [5] found only one case with a GGN component among 47 EML4-ALK-positive lung cancer cases. Fukui et al. [1] also reported only one case with a GGN component among 28 EML4-ALK-positive lung cancer cases, whereas 69 of 140 cases (49.3 %) of EML4-ALK-negative lung cancer included GGN components. Few reports have focused on the imaging findings of EML4-ALK-positive lung cancer. Three of the five reports in Table 1 are proceedings only, and reports include considered both in thin-section CT and in not thin-section CT. Therefore, it is necessary to consider the detailed imaging features of EML4-ALK-positive lung cancer.

The imaging features of EML4-ALK-positive lung cancer are often discussed in relation to their histopathological findings. EML4-ALK-positive lung cancer is common among adenocarcinomas and is associated with an acinar-type histology and sieve-like structure and/or signet ring cells with abundant mucin growing in solid sheets [6]. Therefore, tumors tend to present as solid masses on CT. In the current case, however, mucus-producing cells eventually substituted the alveolar epithelium to produce an irregular tubular-acinar-like structure, resulting in the GGN appearance.

Clinical features of EML4-ALK-positive lung cancer include onset at a younger age and a history of no to light smoking [6, 7]. Additionally, the EML4-ALK fusion gene is mutually exclusive of the EGFR mutation gene [8–10]. One report showed no statistically significant difference in tumor size or lymph node metastasis at the time of diagnosis between EML4-ALK-positive and EML4-ALK-negative cancers [11]. Another study showed no statistically significant difference in overall or progression-free survival between EML4-ALK-positive and EML4-ALK-negative cancers [7]. The same study found that EML4-ALK-positive lung cancer tended to be diagnosed in more advanced stages [7]; however, the present patient showed no difference in her clinical stage for 2 years. Further consideration of the growth speed of EML4-ALK-positive lung cancer is needed; for example, the tumor doubling time may be measured. However, EML4-ALK-positive lung cancer is often highly progressive [9], and careful follow-up is therefore essential in these patients.

## Conclusions

Almost pure GGN had been considered to be less aggressive tumor in lung cancer. In this report, we have shown relatively rare case with potentially aggressive EML4-ALK-positive lung adenocarcinoma showing almost pure GGN. We have to follow this case more carefully than usual.

## Consent

Written informed consent was obtained from the patient for the publication of this case report and accompanying images. A copy of the written consent is available for review by the editor-in-chief of this journal.

## Abbreviations

CT: computed tomography
EGFR: epidermal growth factor receptor
EML4-ALK: echinoderm microtubule-associated protein-like 4-anaplastic lymphoma kinase
GGN: ground-glass nodule
